# A comparison between quantitative PCR and droplet digital PCR technologies for circulating microRNA quantification in human lung cancer

**DOI:** 10.1186/s12896-016-0292-7

**Published:** 2016-08-18

**Authors:** Paola Campomenosi, Elisabetta Gini, Douglas M. Noonan, Albino Poli, Paola D’Antona, Nicola Rotolo, Lorenzo Dominioni, Andrea Imperatori

**Affiliations:** 1 Department of Biotechnology and Life Sciences (DBSV) and “The Protein Factory”, University of Insubria, Via JH Dunant, 3, 21100 Varese, Italy; 2 The Protein Factory, Centro Interuniversitario di Ricerca in Biotecnologie Proteiche, Politecnico di Milano, ICRM-CNR Milano and University of Insubria, Varese, Italy; 3 Department of Surgical Sciences and Human Morphology, DSCM, University of Insubria, Via Guicciardini, 9, 21100 Varese, Italy; 4 Scientific and Technological Pole, IRCCS MultiMedica, Milan, Italy; 5 Department of Public Health and Community Medicine, University of Verona, Verona, Italy

**Keywords:** microRNAs, Lung cancer, Serum biomarkers, qPCR, Droplet digital PCR

## Abstract

**Background:**

Selected microRNAs (miRNAs) that are abnormally expressed in the serum of patients with lung cancer have recently been proposed as biomarkers of this disease. The measurement of circulating miRNAs, however, requires a highly reliable quantification method. Quantitative real-time PCR (qPCR) is the most commonly used method, but it lacks reliable endogenous reference miRNAs for normalization of results in biofluids. When used in absolute quantification, it must rely on the use of external calibrators. Droplet digital PCR (ddPCR) is a recently introduced technology that overcomes the normalization issue and may facilitate miRNA measurement. Here we compared the performance of absolute qPCR and ddPCR techniques for quantifying selected miRNAs in the serum.

**Results:**

In the first experiment, three miRNAs, proposed in the literature as lung cancer biomarkers (miR-21, miR-126 and let-7a), were analyzed in a set of 15 human serum samples. Four independent qPCR and four independent ddPCR amplifications were done on the same samples and used to estimate the precision and correlation of miRNA measurements obtained with the two techniques. The precision of the two methods was evaluated by calculating the Coefficient of Variation (CV) of the four independent measurements obtained with each technique. The CV was similar or smaller in ddPCR than in qPCR for all miRNAs tested, and was significantly smaller for let-7a (*p* = 0.028). Linear regression analysis of the miRNA values obtained with qPCR and ddPCR showed strong correlation (*p* < 0.001).

To validate the correlation obtained with the two techniques in the first experiment, in a second experiment the same miRNAs were measured in a larger cohort (70 human serum samples) by both qPCR and ddPCR. The correlation of miRNA analyses with the two methods was significant for all three miRNAs. Moreover, in our experiments the ddPCR technique had higher throughput than qPCR, at a similar cost-per-sample.

**Conclusions:**

Analyses of serum miRNAs performed with qPCR and ddPCR were largely concordant. Both qPCR and ddPCR can reliably be used to quantify circulating miRNAs, however, ddPCR revealed similar or greater precision and higher throughput of analysis.

**Electronic supplementary material:**

The online version of this article (doi:10.1186/s12896-016-0292-7) contains supplementary material, which is available to authorized users.

## Background

In recent years efforts have been made to find new approaches for early diagnosis of lung cancer, as the prognosis of this disease strongly correlates with stage at the time of diagnosis. Low-dose computed tomography (CT) screening, the only lung cancer screening method endorsed by leading cancer organizations [1–3], has uncertain applicability on a population level as a public health measure, due to high cost, undefined cost/benefit ratio, high false-positive rate, overdiagnosis as well as radiation exposure [4, 5]. Hence, novel methods capable of identifying lung cancer at an early stage are urgently needed.

Recently, microRNAs (miRNAs) have been proposed as a promising class of circulating cancer biomarkers: they are very stable in biofluids and the plasma/serum levels of several circulating miRNAs are aberrantly expressed in many diseases, including numerous cancers [6, 7]. miRNAs are small non-coding RNA molecules of about 20–25 nucleotides that regulate gene expression at the post-transcriptional level [8]. Circulating miRNAs are either stored in particles (exosomes, microvesicles and apoptotic bodies), or associated with RNA-binding proteins or lipoproteins, which prevent their degradation. The abundance and variety of circulating miRNAs suggest a role in cell-cell communication and their potential exploitation as disease biomarkers [9–13]. Among several miRNAs overexpressed in the serum of patients with lung cancer, miR-21 is the most frequently proposed as biomarker of this disease [14–19]. Other miRNAs, that appear to function as tumor suppressors, such as miR-126 and let-7a, are down-regulated in lung cancer patients [20–22].

However, the use of circulating, cell-free, miRNAs as biomarkers for diagnosis of cancer may be proposed only if a reliable and sensitive method for miRNA quantification is available. Quantitative real-time PCR (qPCR) has to date represented the method of choice for this purpose, as it can quantify nucleic acids. Depending on the method used, a “relative” or an “absolute” measurement can be obtained with qPCR, neither of which is devoid of drawbacks. Relative quantification of circulating miRNAs by qPCR, although commonly applied, is hampered by the difficulty of finding reliable endogenous reference genes in the serum, as currently there is no general consensus [23, 24]. On the other hand, absolute quantification is based on calibration curves that are built using known amounts of external calibrators, usually synthetic molecules with the same sequence as the gene of interest. The calibrator concentration however relies on quantification methods and serial dilutions that are themselves prone to uncertainties; moreover, the calibrator amplification kinetics may not reflect that of the endogenous molecules due to differences in matrix composition [25]. Thus, also absolute quantification of a target gene by qPCR is “relative” to the exact quantification of the calibrator [26–29].

Droplet digital PCR (ddPCR) is a recently introduced technology that may facilitate miRNA measurement. The ddPCR technique is based on partitioning of the sample into thousands of micro-reactions of defined volume [30]. After the PCR reaction, each droplet either does or does not contain the nucleic acid of interest, thus allowing estimation of the number of molecules in the reaction under the assumption of a Poisson distribution. Results are expressed as target copies per microliter of reaction [31]. In comparison with qPCR, the ddPCR technique has some favorable features, among which: 1) it performs absolute quantification based on the principles of sample partitioning and Poisson statistics, thus overcoming the normalization and calibrator issues [32]; 2) it has shown increased precision [33] and sensitivity in detecting low target copies [34]; 3) it is relatively insensitive to potential PCR inhibitors [35–37]; 4) it directly provides the result of the analysis expressed as number of copies of target per microliter of reaction (with confidence intervals) [38].

In the present study, carried out at the University of Insubria and the Varese University Hospital, we aimed to compare the performance of the qPCR and ddPCR platforms for quantitative measurement of specific miRNAs in human serum. Three endogenous miRNAs, miR-21, miR-126 and let-7a, which represent candidate biomarkers of human lung cancer [14–22], were examined. We selected these three miRNAs, among numerous miRNAs currently under investigation in many laboratories, for their potential ability to identify individuals with early stage lung cancer [14–22]. An equal number of therapy-naïve, early stage lung cancer patients and age, sex and smoking habit matched controls were included in this study, aiming to explore the performance of the two technologies over the range of miRNA levels to be determined in future studies of human serum samples of interest. Comparison of results of microRNAs expression between patients and controls, as well as analysis of results related to lung cancer detection, are beyond the purpose of the present paper.

### Study design

**Fig. 1:**
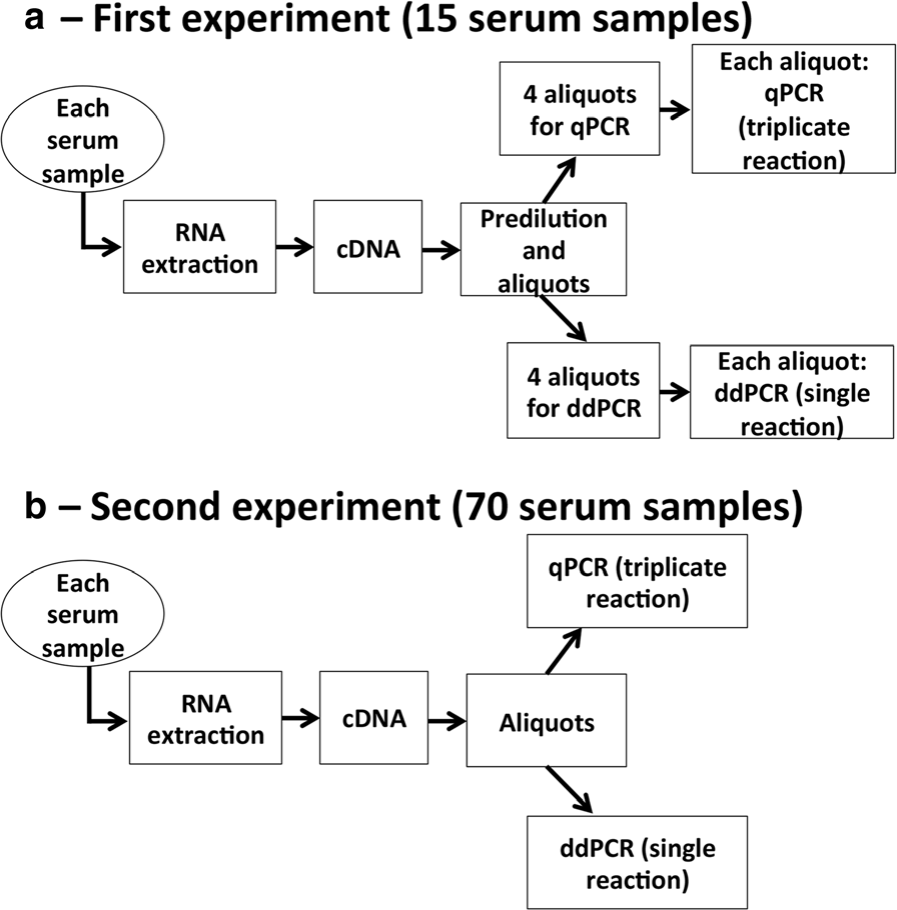
Workflow of the experiments. **a** In the first experiment, for determination of each miRNA of interest (miR-21, miR-126 and let-7a) in each of 15 serum samples, we performed 4 independent qPCRs and 4 independent ddPCRs. **b** In the second experiment, the miRNAs of interest were measured with qPCR and ddPCR in 70 serum samples. All qPCRs were run in triplicate; ddPCRs were run as single reactions

We carried out two experiments. In the first (Fig. 1a), 15 serum samples [7 from patients with early stage lung cancer (stage I and II [39]); 8 from control individuals] were used to compare the precision of miRNA measurements performed with qPCR and ddPCR, and to assess the correlation of measurements obtained with the two methods. In the second experiment (Fig. 1b) we investigated the correlation between the two methods of miRNA analysis in a larger number of samples (35 from early stage lung cancer patients; 35 from controls).

## Results

**Fig. 2:**
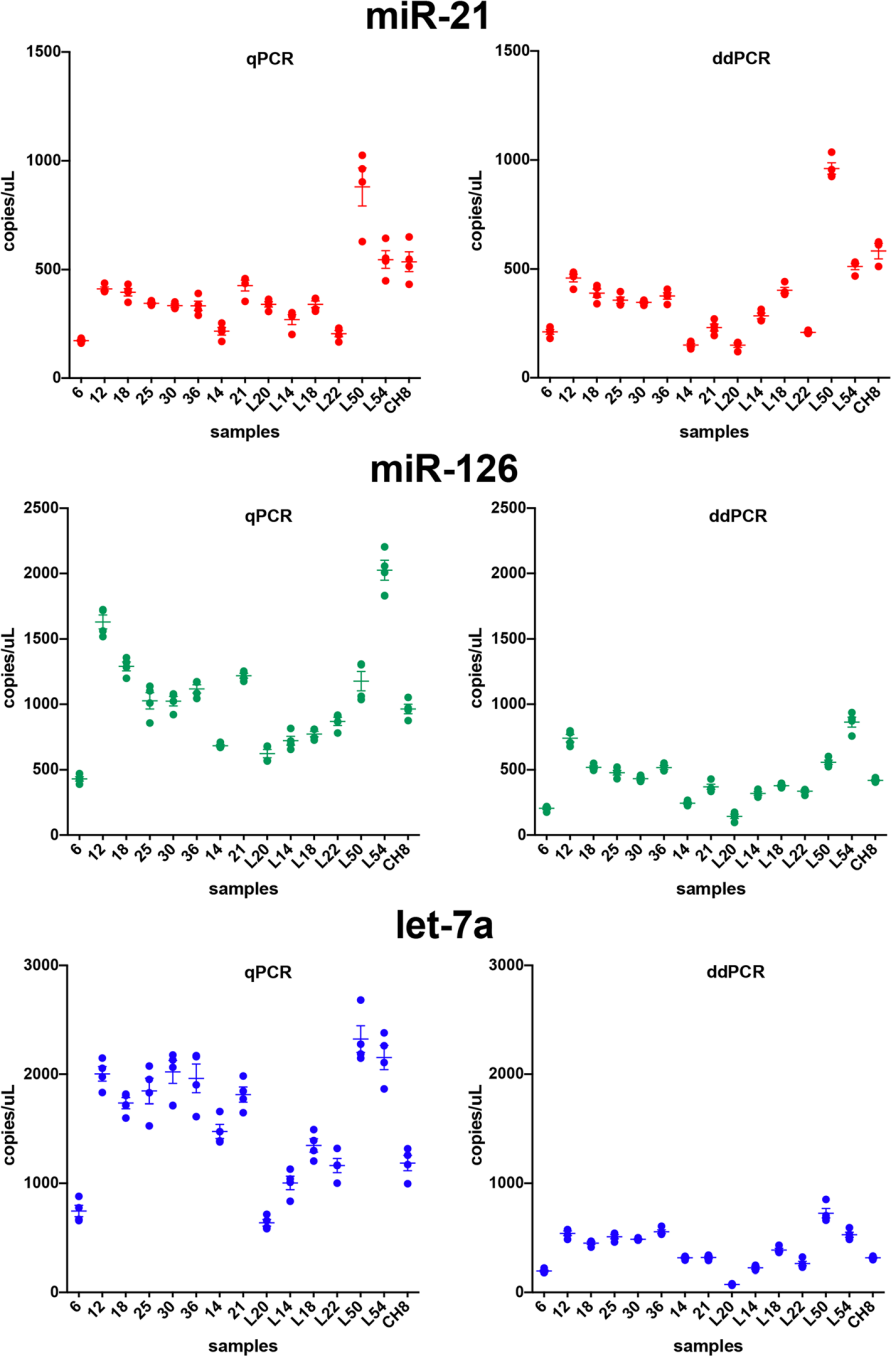
ddPCR frequently shows greater precision compared to qPCR for quantifying miRNAs under study. Scatter plots showing the expression values of the selected miRNAs, by qPCR (*panels on the left*) and ddPCR (*panels on the right*), in the 15 samples (first experiment). Four independent amplifications were performed for each sample in each technique. For qPCR each dot represents the mean of the technical triplicates. The mean and the standard deviation of values obtained from the four independent measurements are shown for each of the 15 samples

In the first experiment (Fig. 1a), four independent analyses for each technique were performed on each cDNA from 15 samples. Each of the four analyses in qPCR was done in triplicate, for a total of 180 amplifications. We found that the trend of expression of miRNAs under study was similar with the two techniques, as can be seen by comparative inspection of scatter plots in Fig. 2. Notably, for miR-126 and let-7a, the samples showed higher values in qPCR as compared to ddPCR: the estimated copy numbers in qPCR were approximately 2.4 and 3.9 fold greater, respectively, than in ddPCR (Fig. 2).

The dispersion of values of the four analyses performed on the same 15 cDNAs was frequently higher with qPCR than with ddPCR (Fig. 2). In the first experiment, the precision of miRNA quantification, measured by the Coefficient of Variation (CV), was significantly better for ddPCR compared to qPCR for let-7a (*p* = 0.028), while it was not significantly different for miR-21 and miR-126 (Table 1).

**Table 1:** Mean and standard deviation of Coefficients of Variation of miR-21, miR-126 and let-7a determinations with qPCR and ddPCR

		n	Coefficient of variation		p*
			Mean	Std. deviation	
Pair 1	miR-21 qPCR	15	11.040	5.4387	0.123
	miR-21 ddPCR	15	8.319	3.2207	
Pair 2	miR-126 qPCR	15	7.386	2.8837	0.675
	miR-126 ddPCR	15	7.944	4.8446	
Pair 3	let-7a qPCR	15	10.298	2.4077	0.028
	let-7a ddPCR	15	7.992	3.2646	

The data for this table were obtained by analyzing 15 samples with four independent analyses with each technique

* paired samples *t*-test

**Fig. 3:**
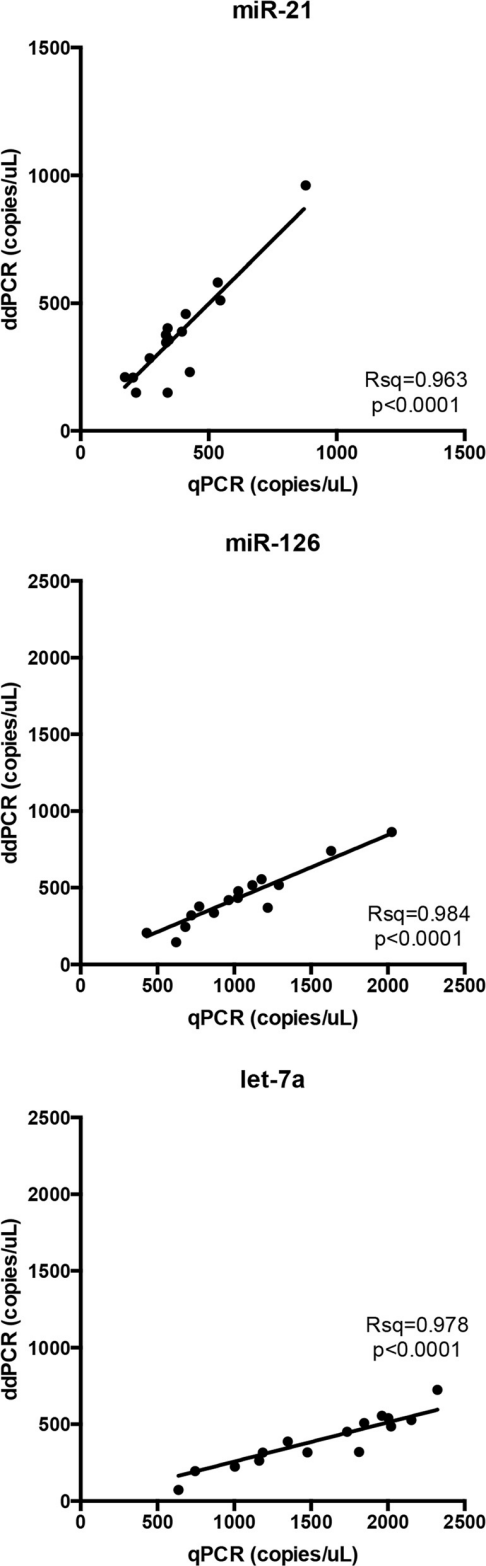
Correlation between qPCR and ddPCR measurements in the first experiment. miR-21, miR-126 and let-7a levels (copies/microliter) were measured in 15 serum samples. Each dot represents the average of 4 independent determinations

Linear regression analysis indicated a significant correlation between qPCR and ddPCR values (Fig. 3). The R-square was 0.963 for miR-21, 0.984 for miR-126 and 0.978 for let-7a (*p* < 0.0001 for all regressions). However, the slope (b) of the regression line for miR-126 (b = 0.420 [CI 95 % 0.391–0.452]) and let-7a (b = 0.2561 [CI 95 % 0.234–0.278]) was significantly lower than one, due to the lower number of copies measured by ddPCR as compared to what estimated with the external calibrator in qPCR.

To verify these systematic differences between qPCR and ddPCR measurements, we quantified by ddPCR the cDNAs of the specific calibrators used to build the calibration curves for qPCR and found a lower concentration than the theoretical one that had been used for calculations in qPCR (data not shown).

**Fig. 4:**
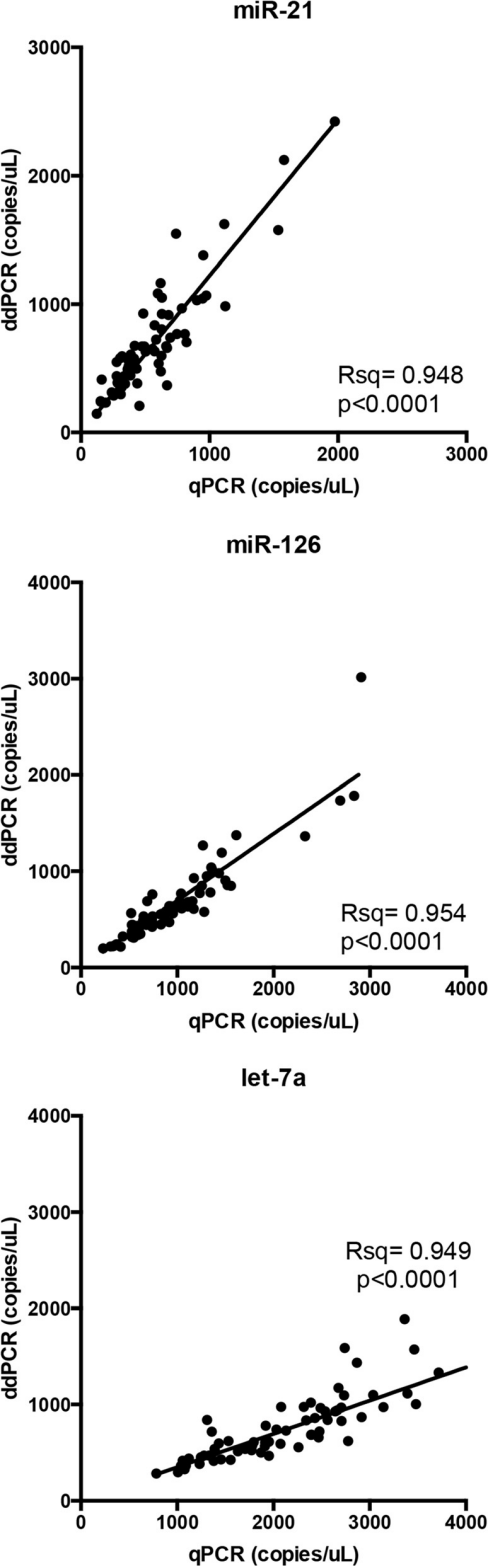
Correlation between qPCR and ddPCR measurements in the second experiment. miR-21, miR-126 and let-7a levels (copies/microliter) were measured in 70 serum samples

In the second experiment, evaluating a cohort of 35 samples from early stage lung cancer patients and 35 from matched controls, again we found significant correlation between qPCR and ddPCR values (R-square = 0.948 for miR-21, 0.954 for miR-126 and 0.949 for let-7a; *p* < 0.0001 for all regressions) (Fig. 4). Consistently, the slopes for miR-126 and let-7a were confirmed to be significantly lower than 1 (b = 0.695 [CI 95 % 0.658–0.731] and b = 0.347 [CI 95 % 0.328–0.366], respectively).

## Discussion

Finding a reliable and sensitive method for quantitation of circulating miRNAs is key to their use as cancer biomarkers. Here we compared the performance of qPCR and ddPCR over the range of miRNA levels to be determined in serum samples of lung cancer patients and controls. For qPCR, the relative quantification method is the most commonly used in the literature, however the lack of reliable endogenous reference miRNAs in biofluids and inability to provide a number of copies of a specific target as the output of analysis, render relative quantification largely unpractical in the perspective of developing a test for clinical use [23].

In qPCR, the absolute quantification method can be used to measure miRNAs of interest in the samples. We estimated the number of target copies in unknown samples based on their Cq compared to that of a standard calibrator. This is a crucial and often underestimated problem, as in qPCR it is assumed that in the calibrator the nominal and measured copies are equivalent in number. The qPCR method relies on accurate quantification of the number of copies of the calibrator, and assumes that no loss of calibrator molecules occurs during the whole procedure. However, errors/imprecisions are possible at several levels [40]: 1) during initial quantification of the standard; 2) during resuspension and preparation of serial dilutions; 3) during reverse transcription (if working on RNA); 4) during amplification itself. Most methods for evaluating nucleic acids concentrations are based on absorbance (optical density), fluorimetric measurements and gel electrophoresis. None of them is devoid of imprecisions; they may not reveal incomplete or partially degraded copies within the sample, or they may still be relative to a standard for accurate quantification [41]. Moreover, calibration standards are often delivered after quantification from the manufacturing company in a dry form, from which the samples need to be resuspended and undergo several dilution steps to reach the concentration useful for calibration purposes. Reverse transcription is a critical step that can account for variability in sample quantification [42]. Also the amplification step can be subject to variability, due to suboptimal efficiency of the assay or to the presence of inhibitors [25–29]. In addition, PCR efficiency of pure synthetic standards may differ from that of serum samples, possibly containing inhibitors [25].

In ddPCR, PCR-positive and PCR-negative droplets are counted at the end of the amplification procedure to directly provide absolute quantification of the target DNA in digital form. The output of analysis is given in copies per microliter of reaction, with 95 % confidence intervals. The ddPCR system allows measurement of DNA copy numbers with remarkable precision; it has been proposed as a method for quantitation of reference materials for the construction of calibration curves [43, 44].

In the present study the results of miRNA analyses with the two techniques significantly correlated. For each of the three miRNAs investigated, the correlation between qPCR and ddPCR measurements was similar comparing the first and the second experiment (Figs. 3 and 4). However, we observed that for miR-126 and let-7a, ddPCR yielded respectively 2.4- and 3.9-fold lower values than qPCR. These discrepancies may be due to a combination of factors, mentioned above, that can affect the qPCR calibration curve. Suboptimal efficiency of retrotranscription, could possibly account for the lower miRNA values estimated by ddPCR [42]. By applying exclusively qPCR to the construction of the calibration curve and to the determination of target concentrations in samples, these inaccuracies would go undetected. The differences we observed in miRNA quantitation between qPCR and ddPCR are comparable with those previously published by other authors [45]. The fact that ddPCR does not require a reference or a standard calibrator curve for quantification, represents one of the main advantages of ddPCR over qPCR.

As for reproducibility of ddPCR analyses, we found high correlation between duplicate ddPCR reactions (R-square = 1; Additional file 1: Figure S1A). Moreover, similar miRNA copy numbers were obtained when samples were measured at different times: immediately after retrotranscription and after storage at −20 °C for an average of 8 months (R-square = 0,979; Additional file 1: Figure S1B). When we compared miRNA measurements by ddPCR after two independent retrotranscription reactions we found a slightly lower reproducibility (R-square = 0.9193; Additional file 1: Figure S1C). When we compared ddPCR measurements of miRNA levels in the same samples analyzed with an identical instrument at another institution we also found similar results (data not shown).

ddPCR averts the need for technical replicates [38], because the sample is partitioned into thousands of micro-reactions. This, in turn, accelerates the quantification process, as more samples, or a higher number of targets, can be analyzed on a single 96 multiwell plate. Recently, instruments and reagents for target quantification based on DNA binding dyes, such as EvaGreen, have become available for digital PCR and have shown results comparable to the use of hydrolysis probes in digital PCR when applied to the quantification of circulating miRNAs [46]. These technological features allowed us to use total cDNA obtained with Exiqon Universal cDNA synthesis kit II and the same Exiqon LNA primer assays to evaluate miRNA expression using both qPCR and ddPCR platforms. Notably, the total cDNA obtained from serum samples can be stored and preserved for future determinations of new potential target miRNAs at later times.

It is important to note that the ddPCR method displayed less variability in replicate analyses than the qPCR method (Fig. 2 and Table 1). It has also been proposed that ddPCR might be less sensitive to differences in sample quality and to the presence of PCR inhibitors, two factors known to influence qPCR amplification efficiency [36, 45, 47]. The fact that ddPCR is an end-point analysis, and that absolute quantification relies on presence or absence of fluorescence in each droplet, rather than the fluorescence levels during the reaction, makes it a robust technique for determining circulating biomarkers. All of these features should simplify the comparison of values obtained with ddPCR assays in different laboratories, while comparison of qPCR results is difficult, as the latter method relies on the use of calibrators or endogenous reference miRNAs, on which there is no consensus. Altogether, we found that the time needed to complete a set of miRNA analyses, including post-PCR processing of data (calculations for qPCR, droplet analysis for ddPCR) was about 2-fold shorter with ddPCR than with qPCR.

Finally, we estimated the direct cost, for the amplification step only, for each miRNA determination in the serum in our laboratory: about 3.33 Euro for qPCR (overall cost for triplicate reactions) and about 3.66 Euro for ddPCR. This cost included all necessary disposables and reagents, starting from cDNA to completion of analysis, while the initial costs for instrument purchase were not taken into account. Thus, in our setting, the price per reaction was very similar with the two techniques.

## Conclusions

We showed a statistically significant linear regression between the quantitative determinations with qPCR and ddPCR of selected circulating miRNAs proposed as lung cancer biomarkers (miR-21, miR-126 and let-7a). Hence, both techniques can reliably be used for quantification of circulating miRNAs as potential biomarkers. Based on the numerous determinations we made for these miRNAs, the recently introduced ddPCR technology compared to qPCR showed similar or greater precision and demonstrated to be a robust method for absolute quantification of selected miRNAs concentration in the serum.

## Methods

### Samples and miRNAs used in this study

Peripheral blood samples (5 mL) were obtained by venipuncture from 70 volunteer adult subjects: 35 therapy-naïve patients with early lung cancer (stage I and II) [39] and 35 age-matched controls (asymptomatic smokers undergoing check-up evaluation). We measured three miRNAs for which a role as cancer biomarkers has been proposed (miR-21; miR-126; let-7a).

### Comparison of qPCR and ddPCR

To compare the two methods of miRNA quantification, we extracted total RNA and prepared cDNA starting from each serum sample, obtained as outlined above. To ensure that intersample variability was kept to the minimum, we worked using constant volumes throughout the whole procedure, starting from serum to the final amplification step.

### First experiment

To compare the precision of miRNA measurements performed with qPCR and ddPCR, and to assess the correlation of the measurements obtained with the two methods, cDNA from each of the 15 samples was divided into aliquots that were used to perform four independent qPCR and four independent ddPCR analyses, on different days. Each qPCR analysis was done in triplicate and the average of the triplicate values represents every single result of the qPCR analysis. For ddPCR, the analysis of each miRNA in each sample was done as single reaction (Fig. 1a). All data were expressed as copies/μL of reaction. Copies were extrapolated from the standard curves for qPCR (see below) or directly represented the output of the QuantaSoft software for ddPCR. However, also in the latter case, manual inspection of the samples was performed to set the fluorescence threshold and divide droplets not containing from droplets containing the template. For each of the 15 samples, the following three miRNAs were analyzed: miR-21, miR-126 and let-7a, along with the relative quality controls (spike-ins), to check for extraction and retrotranscription efficiencies and presence of potential inhibitors, as well as hemolysis indicators (miRNAs deriving from lysis of red blood cells that may influence the measured value).

### Second experiment

Using both qPCR and ddPCR we quantified the level of miR-21, miR-126 and let-7a in the serum samples of 35 patients with early stage lung cancer and 35 controls. In this experiment a single analysis was performed for each technique (in qPCR the value used for analyses is the average of three technical replicates) and for each of the three miRNAs of interest in each serum sample (Fig. 1b). Quality control miRNAs were analyzed as described in the first experiment.

### Serum preparation and RNA extraction

Peripheral blood was collected using sterile tubes without anticoagulant and left at room temperature to coagulate for a minimum of 30 and a maximum of 60 min. Then serum was separated by centrifugation at 400 g for 8 min at 4 °C, was divided in 500 μL aliquots and stored at −80 °C until total RNA extraction.

Purification of total RNA, including miRNAs, was performed using the miRNeasy serum/plasma kit (Qiagen, Milan, Italy), starting from 200 μL of serum and following manufacturer’s instructions. One μg of MS2 phage carrier RNA (Roche, Monza, Italy) and 1 μL of a mix of UniSp2, UniSp4 and UniSp5 spike-ins (Exiqon, Euroclone, Milan, Italy) were added just before the purification process to assess efficiency of RNA purification and presence of possible PCR inhibitors. RNA was eluted from the column with 14 μL of nuclease-free water and stored at −80 °C.

### Retrotranscription

Two μL of RNA were reverse transcribed to cDNA in 10 μL total reaction, using the Universal cDNA synthesis kit II, as part of the miRCURY LNA™ Universal RT microRNA PCR system (Exiqon, Euroclone, Milan, Italy) according to the manufacturer’s instructions. For some samples also a “no enzyme” control reaction was prepared and subsequently analyzed by qPCR in parallel with their retrotranscribed counterparts, to check for “non miRNA specific” amplification. 0.5 μL of UniSp6 and cel-miR-39-3p spike-ins were added when assembling the reactions for subsequent evaluation of efficiency of the reverse transcription step. For the first experiment, conducted on 15 serum samples, the cDNA was prediluted 4-fold, split into four aliquots of 5.6 μL for qPCR and four aliquots of 3 μL for ddPCR and stored at −20 °C until use; this was made to avoid repeated freeze and thawing of the cDNAs. For the second experiment, conducted on 70 samples with single analyses, the cDNA samples were stored at −20 °C undiluted.

### Real-time qPCR

The cDNAs were diluted further 10 fold from the predilutions (first experiment, 15 samples) or 40 fold from undiluted reactions (second experiment, 70 samples) just before use. Four microliter were used in each 10 μL qPCR reaction, completed with the addition of 6 μL of reaction mixture, composed of 1 μL of the specific miRCURY LNA PCR primer set and 5 μL of ExiLENT SYBR Green master mix (both from Exiqon-Euroclone, Milan, Italy). All reactions were performed in triplicate and the values shown are means of these replicates (Fig. 1) A no template control (NTC), where distilled water was added instead of cDNA samples, was included for each reaction mix prepared. A CFX96 realtime PCR instrument (Biorad, Milan, Italy) was used, following the manufacturer’s instructions for cycling conditions [95 °C for 10 min, followed by 40 cycles of 95 °C for 10 s and 60 °C for 1 min (1.6 °C/s ramp rate)].

The samples were monitored for hemolysis by calculating the difference between the Cq values of miR-23a and miR-451a, evaluated by qPCR, according to the Exiqon guidelines. Samples were considered at risk of hemolysis when their ΔCq (Cq_miR-23a_ - Cq_miR-451a_) was > 5.

### Standard curve construction and sample absolute quantification with qPCR

Three unmodified oligoribonucleotides corresponding to hsa-miR-21-5p (UAGCUUAUCAGACUGAUGUUGA), hsa-miR-126-3p (UCGUACCGUGAGUAAUAAUGCG) and hsa-let-7a-5p (UGAGGUAGUAGGUUGUAUAGUU) were synthesized (Eurofins Genomics, Milan, Italy). Different dilutions of oligoribonucleotides in RNase-free water were prepared and the appropriate dilution (6 × 10^4^ copies) was reverse transcribed to cDNA in 10 μL total reaction, using the Universal cDNA synthesis kit II, as part of the miRCURY LNA™ Universal RT microRNA PCR system (Exiqon, Euroclone, Milan, Italy). A two-fold dilution series over nine points were prepared from the cDNA, starting from a dilution at 2000 copies/μL, then the nine dilutions were used as templates for qPCR. Each point was performed in triplicate. The standard curve was constructed by plotting Cq values against the logarithmic concentration of the calibrator oligoribonucleotides. The amount of an unknown sample was quantified by interpolating the Cq values in the standard curve.

### Droplet digital PCR

The cDNAs were diluted as described in the previous section and 5 μL were used in each ddPCR reaction, adding the desired miRCURY LNA PCR primer set at the appropriate dilution (Table 2), experimentally determined by testing two different volumes of primers, 10 μL of QX200 EvaGreen ddPCR Supermix (Biorad, Milan, Italy) and nuclease-free water up to 20 μL. A no template control (NTC), where distilled water was added instead of cDNA samples, was routinely included for each reaction mix prepared. Each 20 μl ddPCR reaction was loaded into an 8-channel droplet generation cartridge (Biorad, Milan, Italy); 70 μL of QX200 Droplet generation oil (Biorad, Milan, Italy) was added into the appropriate wells and the cartridge was loaded in the QX200™ Droplet Generator (Biorad, Milan, Italy) to generate the emulsion. The resulting droplets were transferred to a 96-well plate (Eppendorf) with a Rainin multichannel pipette, the plate sealed with Pierceable foil (Biorad, Milan, Italy) and amplified by standard PCR using a Mastercycler® ep (Eppendorf). Cycling conditions were: 95 °C for 5 min, followed by 40 cycles of 95 °C for 30 s and 60 °C for 1 min, followed by signal stabilization steps (4 °C for 5 min, 90 °C for 5 min) and final hold at 4 °C. The ramp rate was 2 °C/s. After PCR, plates were loaded into QX200™ Droplet Reader (Biorad, Milan, Italy) for detection.

**Table 2:** Primer sets used in the study and volumes for ddPCR

Candidate miRNAs under study	Exiqon ID	Volume used in ddPCR (microliters)
miR-21-5p	204230	1
miR-126-3p	204227	0.5
let-7a-5p	205727	1

The resulting copies per microliter of reaction were adjusted depending on the number of microliters of cDNA used in qPCR and ddPCR before making comparisons between the two techniques.

### Statistical analyses

Correlation between the qPCR and the ddPCR output analyses was tested by linear regression model. The precision of miRNA measurements was estimated with the Coefficient of Variation [CV = (SD/mean)*100] of quadruplicate measures for each sample, for both qPCR and ddPCR. The CVs of the two assays were compared by *t*-test for paired data. A *p* value <0.05 was considered statistically significant.

Data were analyzed with SPSS 10.6 software (Illinois, USA).

## Additional file

Additional file 1: Figure S1. Reproducibility of miR-126 quantification by ddPCR: in 10 cDNA samples analysed in duplicate on the same day (A); in 8 cDNA samples before and after storage at −20 °C for 8 months (B); in 4 samples after two independent retrotranscription reactions (C). (TIF 100 kb)

## Abbreviations

CV: Coefficient of variation
ddPCR: Droplet digital PCR
miRNA(s): microRNA(s)
qPCR: Quantitative real-time PCR
