# Supplementary figures and images for: A comparison between quantitative PCR and droplet digital PCR technologies for circulating microRNA quantification in human lung cancer
Source: BMC Biotechnol. 2016 Aug 18;16:60. doi: 10.1186/s12896-016-0292-7 (PMC4991011; doi:10.1186/s12896-016-0292-7)

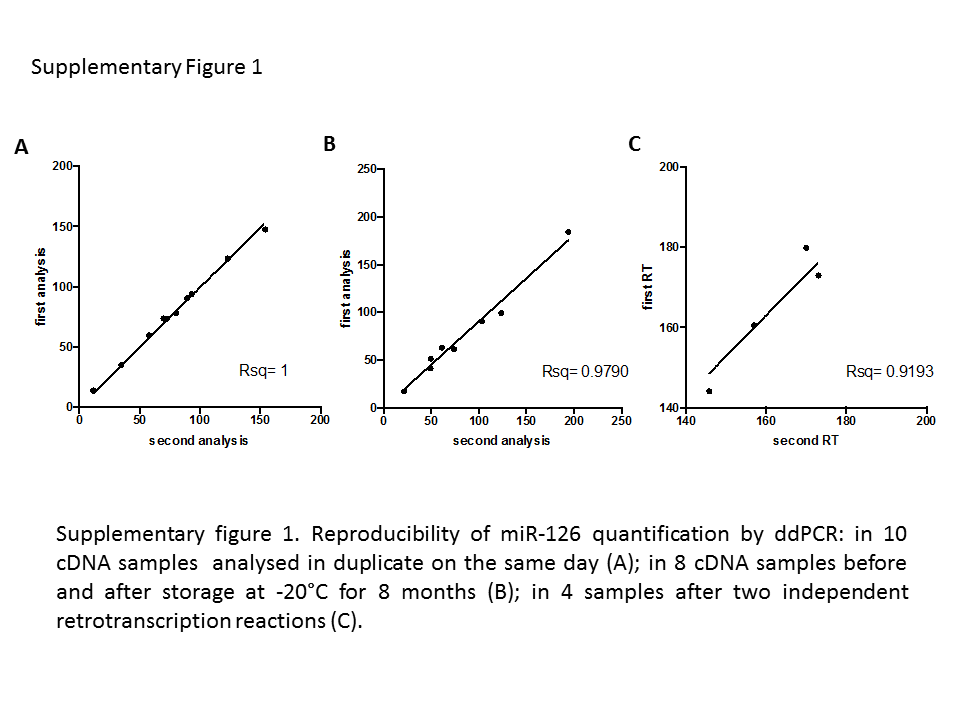

Supplement: Additional file 1: Figure S1. — Reproducibility of miR-126 quantification by ddPCR: in 10 cDNA samples analysed in duplicate on the same day (A); in 8 cDNA samples before and after storage at −20 °C for 8 months (B); in 4 samples after two independent retrotranscription reactions (C). (TIF 100 kb) [file 12896_2016_292_MOESM1_ESM.tif]
